# The 2018 Ming K. Jeang award for excellence in *Cell & Bioscience*

**DOI:** 10.1186/s13578-019-0305-z

**Published:** 2019-05-20

**Authors:** Yun-Bo Shi

**Affiliations:** The National Institutes of Health, Bethesda, USA

## Abstract

Two research papers, one from a group led by Dr. Haifan Lin of Yale University School of Medicine, New Haven, USA and another from two laboratories led by Drs. Yihong Ye of National Institute of Diabetes and Digestive and Kidney Diseases, National Institutes of Health, Bethesda, USA, and Ting Zhang of Southern University of Science and Technology, Shenzhen, China, respectively, won the 2017 Ming K. Jeang Award for Excellence in *Cell & Bioscience.*

## Editorial

We are very pleased to announce that two research articles published in *Cell & Bioscience* in 2018 have been selected to receive the Ming K. Jeang Award for Excellence in *Cell & Bioscience*. The Ming K. Jeang Award for Excellence in *Cell & Bioscience* was established in 2011 with a generous donation from the Ming K. Jeang Foundation to honor outstanding research articles published in *Cell & Bioscience*, the official journal of the Society of Chinese Bioscientists in America (SCBA; http://www.scbasociety.org). A committee of *Cell & Bioscience* Editors, chaired by Dr. Dong-Yan Jin, considered all research articles published in the journal in 2018 to select the following two articles to receive the award [1, 2]:

## A role of Pumilio 1 in mammalian oocyte maturation and maternal phase of embryogenesis

Winifred Mak, Jing Xia, Ee-Chun Cheng, Katie Lowther and Haifan Lin

Cell & Bioscience 2018 8:54.

## Proteomic characterization of endogenous substrates of mammalian ubiquitin ligase Hrd1

Yilin Ye, Suk-Hwan Baek, Yihong Ye and Ting Zhang

Cell & Bioscience 2018 8:46.

Congratulations to these two groups of investigators for jobs well done!

We are looking forward to receiving contributions of outstanding research articles from the scientific community in 2019 and beyond.
